# Electronic Health Record-Based Screening for Major Cancers: A 9-Year Experience in Minhang District of Shanghai, China

**DOI:** 10.3389/fonc.2019.00375

**Published:** 2019-05-22

**Authors:** Dandan He, Wanghong Xu, Hualin Su, Weixi Li, Jie Zhou, Baodong Yao, Dongli Xu, Na He

**Affiliations:** ^1^Center for Disease Control and Prevention of Minhang District, Shanghai, China; ^2^Department of Epidemiology, School of Public Health, Fudan University, Shanghai, China; ^3^Key Laboratory of Public Health Safety, Ministry of Education, Fudan University, Shanghai, China

**Keywords:** electronic health record, cancer screening, health management, major cancer, information platform

## Abstract

**Background:** An electronic health record (e-HR) system has been developed in Minhang District of Shanghai, China, since 2005, making it convenient for local health institutions to provide integrative and comprehensive health care and management for major diseases.

**Methods:** In 2008, an e-HR-based cancer prevention program was initiated to screen multiple cancers, including colorectal, gastric, liver, lung, cervical, and breast cancers, and provide subsequent health education and health management to cancer patients and high-risk individuals. This study was designed in prospective analysis, based on the constructive analysis of key information, observation of cancer screening and healthcare processes and organizations, and stages of cancers detected by the e-HR-based programs.

**Results:** From 2008 to 2016, health education was conducted for over 5 million attendances, and more than 3 million screening tests were performed for eligible residents over 40 years old. A total of 2,948 cancer cases were detected, accounting for 13.3% of all newly diagnosed cancers in the district during the 9-year period. Thirty point seven percent detected cancer cases were at the early stage, significantly higher than the 22.9% in cases identified by e-HR-based follow-up and 13.8% in cases diagnosed due to signs or symptoms. More than 136,000 residents were identified as individuals at high risk of cancer and subject to sustainable clinical follow-up and health management.

**Conclusions:** The successful application of e-HR system in cancer prevention and control in Minhang district of Shanghai, China, implies that the system may act as an extendable and sustainable infrastructure for comprehensive health care and services for a broad spectrum of diseases and health events.

## Introduction

**Figure 1:**
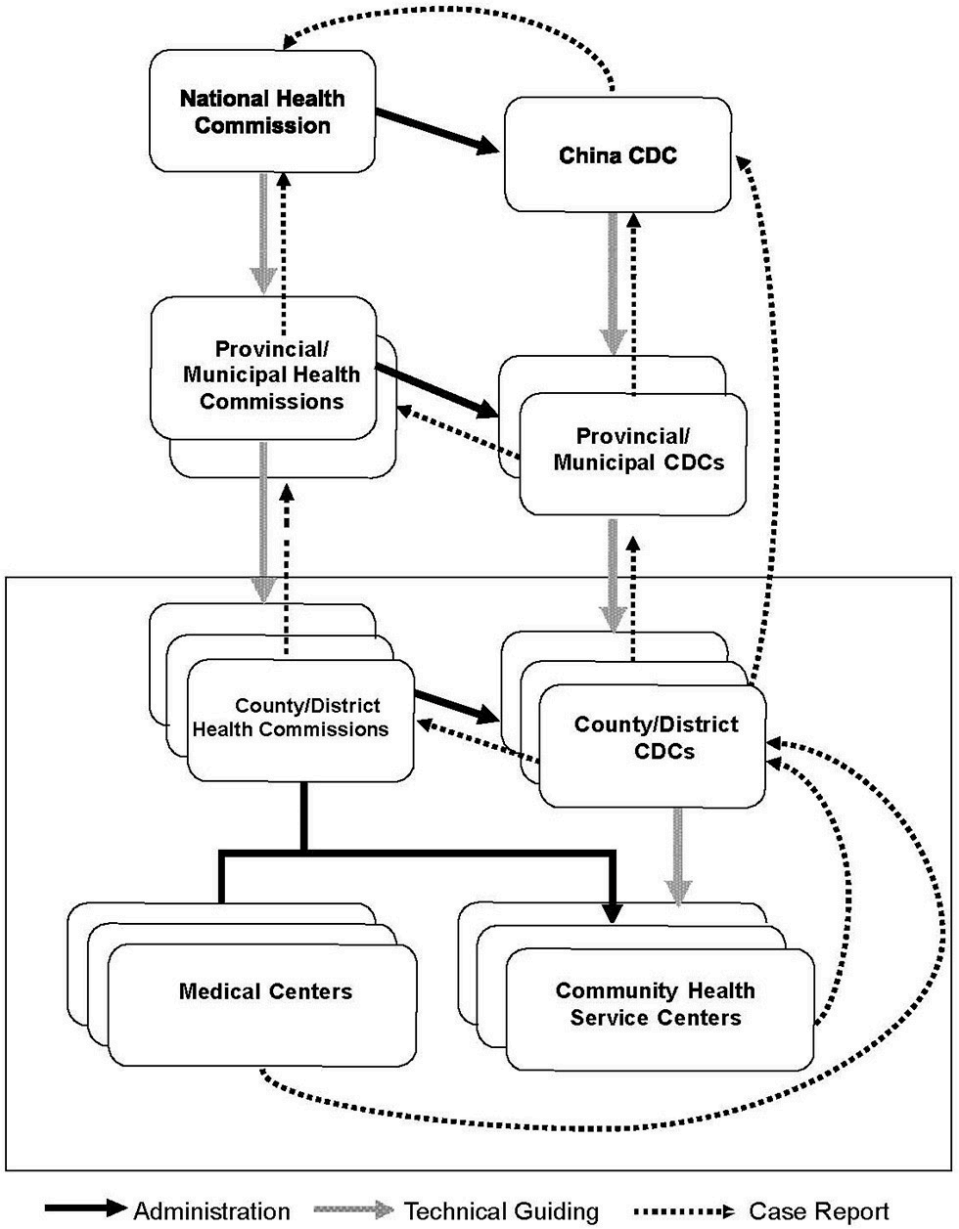
Institutional infrastructure of disease control system and cancer care services in China. Frame: Functional units of county/district-level institutions that practice the basic services.

In China, a vertical networking system for non-communicable disease prevention and control has been well-established, in which national, provincial, municipal, and local Health Commissions; the Centers for Disease Control and Prevention (CDC); offices for specific disease control; hospitals; and Community Healthcare Service Centers (CHSC) are supposed to work together to fight against non-communicable diseases (1). At the county/district level, a tree-like structured healthcare system was also established, with local CDC, CHSC, and medical centers working as functional units for comprehensive healthcare services under the administration of the local Health Commission (Figure 1). Due to lacking health information sharing platform, however, health information and health records could not be exchanged and shared among these executive institutions. As a result, health services offered by different institutions, from primary to tertiary, were not effectively integrated.

Cancer care provided at the county/district level is a typical example of separated public service delivery: health education and screening programs are usually organized by local CDCs, while clinical diagnosis and treatment of cancers are performed by local medical centers and community-based check-up and post-treatment services are provided by CHSCs (2). Due to a lack of a referral system, these institutions remain distinct and independent from each other. Only in limited areas where the cancer registry system is well-established will medical centers report newly diagnosed cancer cases to a local cancer registry system, from which the local CDC and CHSCs can be alerted of occurrence of the disease and then provide standardized health care to the patients (3). However, this happens in the absence of detailed feedbacks and technical supports from medical centers for specific and individualized patient care.

To solve the problem, Minhang District, one of the 18 administrative districts in Shanghai, China, established an electronic health record (e-HR) system in 2005. The system has been used to comprehensively deliver various health services and improve accessibility and quality of services (4). In 2008, Minhang district initiated a comprehensive cancer screening program based on the e-HR system (5), aimed to improve the effectiveness and efficacy of cancer screening with a seamless interface of government machinery.

In this article, we introduced the 9-year experience of the e-HR-based cancer screening and health management for detected cancer cases and individuals at high risk and thus provide recommendations for seamless service delivery in the real world.

## Materials and Methods

This study was chosen as a prospective design, based on the constructive analysis of key information, organizations, and observations of cancer screening, stages of cancers detected by the e-HR-based programs, and healthcare processes for high-risk population.

The study material was the process and results of the e-HR-based screening programs for six major cancers, i.e., colorectal cancer, gastric cancer, liver cancer, lung cancer, cervical cancer, and breast cancer, among residents in Minhang District of Shanghai, China. All data for this study were extracted from the established e-HR system. The study was approved by the Institutional Review Board of Minhang District CDC (NO: EC-P-2012-002). Verbal informed consent was obtained from each participant of the cancer screening program.

### Electronic Health Record System in Minhang District

**Figure 2:**
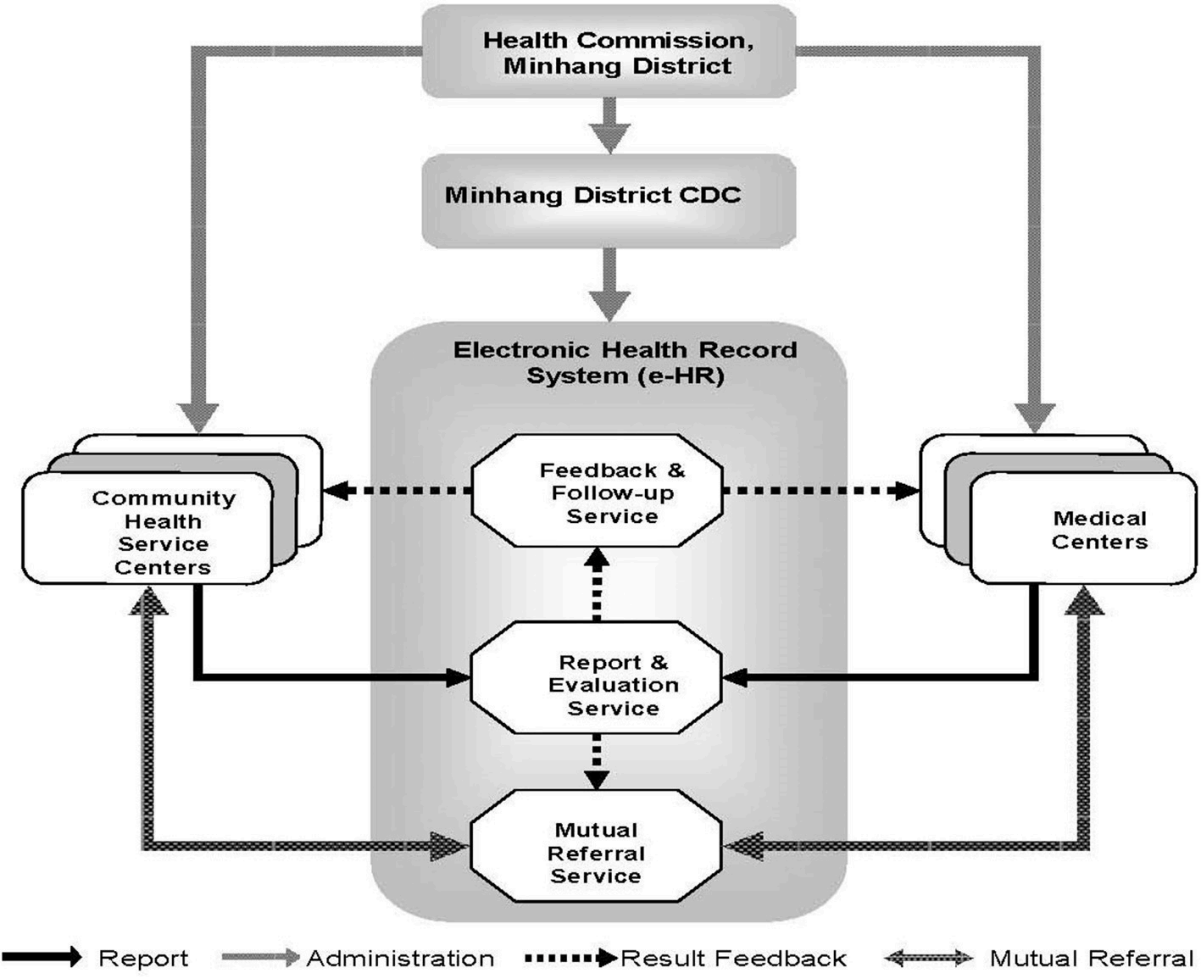
Administrative and institutional infrastructure of electronic health record (e-HR)-based early detection and continuous health management for six major cancers in Minhang district of Shanghai, China.

The Minhang e-HR system is a comprehensive information platform integrating primary care, medical or clinical records, vaccine inoculation, and other public health activities (Figure 2). It is administrated by the Health Commission of Minhang District and executed by the CDC of Minhang District. All CHSCs and medical centers in the district were organized and interlinked within the system.

All residents in Minhang District have a medical care card implanted with a microchip, through which their medical records and healthcare information are recordable and accessible by responsible doctors in all local health institutions, including CHSCs and medical centers in the district. The information is also accessible by staff in CDC by logging in the e-HR system. There are three major functions of the e-HR system: (1) to report and evaluate medical records, (2) to feedback clinical results and conduct follow-up, and (3) to provide mutual referral service.

The Minhang e-HR system was established in 2005 and covered 93.05% (*n* = 830,400) of the local permanent residents and 30.5% (*n* = 334,800) of a migrant population in 2014 (6). So far, the e-HR system has included information for over 2.6 million people, covering almost all residents in the district.

### Comprehensive Electronic Health Record-Based Cancer Prevention Programs

The cancer prevention programs in Minhang District are a series of comprehensive healthcare services provided based on the established e-HR system. Three major modules were included in the e-HR system to promote accessibility and implementation of the early detection of cancers: (1) health education on cancer prevention. Usually, training courses on cancer prevention were delivered by general practitioners in CHSCs for residents aged over 40 years. These residents were asked to record their attendance in any courses in the e-HR system by scanning their medical care card; (2) free health check-up programs, including health check-up for senior residents over 60 years, “two cancer” (breast and cervical cancer) screening for vulnerable women population, and health check-up for rural residents. The subjects could be identified through the e-HR system; and (3) opportunistic screening in all local clinics and medical centers. Physicians involving in the program were reminded by the e-HR system automatically to provide free cancer screening for those who had certain risk factors, related symptoms, or claims.

### Application of Electronic Health Record System in Cancer Risk Assessment

In all health institutions, the recruited residents were asked to answer whether they had (1) cancer-related symptoms like changed shape/property of feces, phlegmatically blood feces, abdominal pain, hematemesis, anemia, cough or expectoration sputum, abnormal vaginal secretions (women only), abnormal nipple discharge (women only), etc.; (2) precancerous lesions, such as digest duct polyps, adenomas, gastric intestinal metaplasia, atrophic gastritis, or cervical intraepithelial neoplasia (women only), etc.; (3) family history of the six major cancers; (4) occupational exposures to radon, arsenic, chromium, nickel, or asbestos; (5) cigarette smoking; (6) infections with HBV, HCV, HPV, or other cancer-related pathogens; (7) infertility (women only); and (8) use of estrogens or oral contraceptives (women only).These information were entered into the e-HR system, based on which individuals' risks for the six major cancers could be evaluated by all health institutes involved according to the criteria predefined based on the guidelines (7, 8) or previous studies (9–15).

### Application of Electronic Health Record System in Cancer Screening

Individuals with positive results in risk assessment were referred to receiving respective free initial screening tests. The testing results, both negative and positive, were entered into the e-HR system by staffs in CHSCs and could be accessed by staff in Minhang CDC through logging in the system and by doctors in secondary or tertiary medical centers using patients' medical care cards implanted with a microchip.

### Referral, Follow-Up, and Information Exchange

A mutual referral and information exchange mechanism between CHSCs and secondary or tertiary medical centers was also established within the framework of e-HR system (Figure 2). For example, if a resident was negative in cancer screening tests but evaluated to be at high risk of cancer, he/she would be referred to secondary or tertiary medical centers in Minhang District for further clinical examinations. On the other hand, if a resident was positive in cancer screening tests, he/she would be visited by a CHSC doctor within 1 month for community-based primary care and a physician in medical centers for clinical follow-up and medical care. The whole process and results of questionnaire-based risk assessment, screening, community and clinical follow-up, and health management were electronically recorded and centralized in the district-level database and were available for all health institutions in the district.

For newly diagnosed cancer cases, an effective referral mechanism ensures information exchange between local and municipal Cancer Registry System. Once a permanent resident in Minhang District was diagnosed with cancer in hospitals in the district, his/her information would be reported to the local Cancer Registry System first and then to the Shanghai Municipal Cancer Registry. On the contrary, if a permanent resident of Minhang District was first diagnosed with cancer outside the district, his/her information would be reported to the Shanghai Municipal Cancer Registry System first and then was recognized and added to the e-HR database and local Cancer Registry by the CDC of Minhang District. Thereby, the information of all cancer cases could be accessible for designated continuous clinical follow-up and health management by local institutes.

### Continuous Health Management for High-Risk Individuals

**Figure 3:**
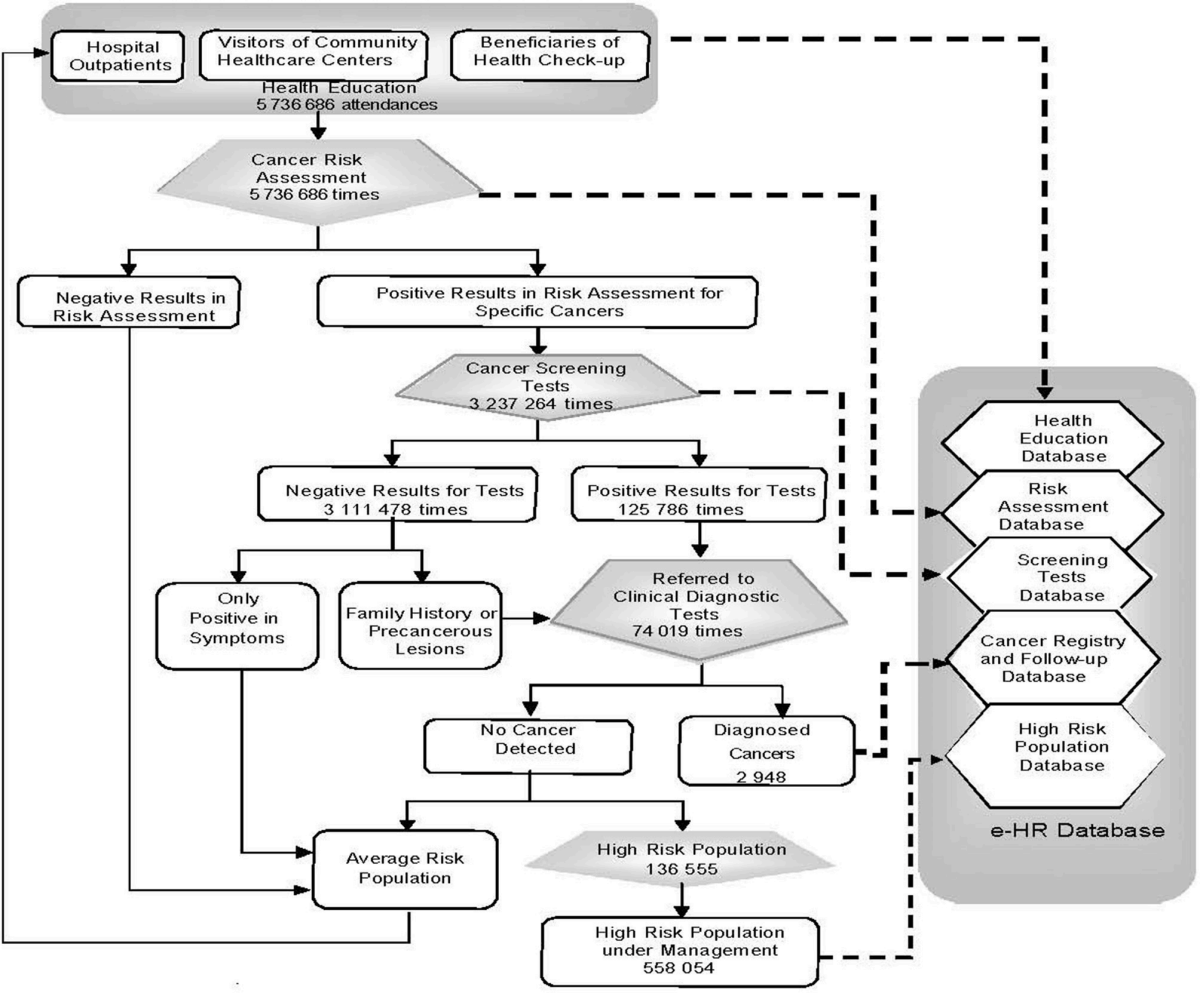
Reporting process of e-HR-based early detection for six major cancers in Minhang District of Shanghai, China, 2008–2016. Solid arrow: resident flow; dashed arrow: information flow.

Targeted residents who met either of the following two criteria were identified as high-risk individuals: (1) with a positive result in initial screening tests but a negative result in diagnostic tests and (2) with a family history of any cancer or a precancerous lesion. For this population, doctors at CHSCs were designated to provide primary care and follow-up services per month, which include health education, behavioral interventions and routine clinical tests, and if necessary, referral advices for further qualified diagnosis and clinical care in medical centers (Figure 3).

### Data Analysis

Descriptive analyses were performed by presenting the number and percentage of residents in each subgroup. Chi-square tests were used to compare proportions of early-stage cancers among those screen-detected, identified by follow-up or diagnosed by clinic visits. The trend analysis was conducted by using *p*-values for row mean score differences in Cochran–Mantel–Haenszel statistics. A *p-*value of < 0.05 was considered as statistically significant. SPSS 11.0 software was used in all data analyses.

## Results

Table 1 presents the criteria to identify eligible subjects for initial cancer screening tests based on e-HR system. All residents over 40 years in Minhang district were eligible for colorectal cancer and gastric cancer screening programs. For liver cancer and lung cancer screening, only those with positive results in risk assessment were recruited. For cervix uteri cancer and breast cancer screening, all women over 40 years receiving free health check-up were included, while only those with positive results in risk assessment were recruited in CHSCs and medical centers.

**Table 1:** Criteria used to define high-risk individuals in electronic health record (e-HR)-based risk assessment in Minghang District of Shanghai, China.

**Cancer site**	**Criteria used to define high-risk individuals**	
	**In CHSCs and medical centers**	**In health check-up**
Colorectum	Over 40 years	
Stomach	Over 40 years	
Liver	Positive in risk assessment^†^	
Lung	Positive in risk assessment^†^	
Cervix uteri ^‡^	Positive in risk assessment^†^	Over 40 years
Breast ^‡^	Positive in risk assessment^†^	Over 40 years

*CHSC, Community Healthcare Service Center*.

† *With related family history, risk behaviors, or symptoms*.

‡ *Only for women*.

As shown in Table 2, subjects identified with high risk of respective cancers received fecal occult blood test (FOBT) and/or rectal exam as initial screening tests for colorectal cancer, had FOBT for gastric cancer, alpha-fetoprotein (AFP) and ultrasonic testing for liver cancer, chest X-ray for lung cancer, Pap smears for cervix uteri cancer, and clinical breast examination (CBE) and thermal texture maps (TTMs) for breast cancer. These tests, as well as tumor-associated antigen test, mammography, low-dose computerized tomography, colonoscopy, gastroscopy, or colposcopy, were further provided for those with negative results in diagnostic tests or those with a family history of cancer or precancerous lesion as a continuous health management service.

**Table 2:** Initial screening tests used in e-HR-based cancer screening programs in Minhang District of Shanghai, China.

**Cancer site**	**Initial screening tests used in subgroups**			**Continuous health management services^†^**
	**Visitors of CHSCs**	**Outpatients in medical centers**	**Beneficiaries of health check-up**	
Colorectum	FOBT	FOBT + RE	FOBT	Colonoscopy^‡^
Stomach	FOBT	FOBT	FOBT	Gastroscopy
Liver	AFP + UT	AFP + UT	AFP + UT	AFP + UT
Lung	Chest X-ray	Chest X-ray	Chest X-ray	Chest X-ray or LDCT
Cervix uteri	Pap smear	Pap smear	Pap smear	Pap smear + colposcopy
Breast	CBE + TTM	CBE + TTM	CBE + TTM	MAM or UT

*CHSC, Community Healthcare Service Center; FOBT, fecal occult blood test; RE, rectal exam; AFP, alpha-fetoprotein; UT, ultrasonic testing; LDCT, low-dose computerized tomography; CBE, clinical breast examination; TTM, thermal texture maps; MAM, mammography*.

† *Only for individuals with a negative result in diagnostic tests or with a family history of any cancer or having a precancerous lesion*.

‡ *FOBT or RE for some subjects*.

During the period of 2008 to 2016, the proportion of targeted residents receiving questionnaire-based risk evaluation increased from 3.1 to 22.2% for colorectal cancer (*p* for trend < 0.01), from 1.6 to 23.0% for gastric cancer (*p*-trend < 0.01), from 7.3 to 20.5% for liver cancer (*p* trend < 0.01), and from 1.8 to 21.2% for lung cancer (*p* trend < 0.01) (Table 3). The proportion also increased for cervix uteri cancer and breast cancer from 2008 to 2014 but decreased in 2015.

**Table 3:** Cancers and high-risk individuals identified in e-HR-based cancer screening programs in Minhang District of Shanghai, China, 2008–2016.

**Cancer site**	**Calendar year**	**All subjects**		**Participants of e-HR-based cancer screening programs**						
		**No. of eligible residents**	**No. of incident cancers**	**% of participants for risk assessment**	**No. of subjects for screening tests**	**No. of subjects with positive results**	**Cancer cases detected**	**Proportion of detected cancers (%)**	**No. of high-risk individuals**	No. of high-risk individuals receiving health management^**^
**COLORECTUM**										
	2008	491,853	476	3.1	15,351	924	34	7.1	1,151	–
	2009	511,366	567	18.6	76,407	1,647	84	14.8	3,225	1,065
	2010	469,691	569	24.6	115,630	2,854	119	20.9	1,838	3,574
	2011	549,318	527	21.3	117,039	1,883	98	18.6	1,337	7,765
	2012	563,525	512	34.4	193,654	5,678	164	32.0	4,260	11,153
	2013	577,892	639	32.3	186,391	7,350	183	28.6	5,075	10,800
	2014	592,322	598	29.5	174,581	6,888	160	26.8	3,109	20,409
	2015	606,961	640	22.2	134,790	6,181	124	19.4	2,322	21,555
	2016	623,788	628	22.2	138,445	8,861	112	17.8	1,961	18,446
**STOMACH**										
	2008	491,853	417	1.6	8,035	206	12	2.9	924	–
	2009	511,366	393	15.5	79,342	781	49	12.5	1,198	686
	2010	469,691	403	25.6	120,104	2,190	86	21.3	1,402	3,634
	2011	549,318	385	22.3	122,557	1,522	73	19.0	1,126	8,251
	2012	563,525	331	36.2	203,723	4,000	106	32.0	2,325	9,697
	2013	577,892	454	33.6	194,343	5,234	126	27.8	1,862	18,485
	2014	592,322	420	30.6	181,057	5,190	127	30.2	1,676	23,261
	2015	606,961	462	23.4	142,288	4,552	65	14.1	1,127	11,200
	2016	623,788	483	23.0	143,236	8,619	84	17.4	1,863	10,462
**LIVER**										
	2008	491,853	251	7.3	15,181	52	14	5.6	481	–
	2009	511,366	242	16.4	3,448	15	9	3.7	2,152	291
	2010	469,691	280	24.0	6,376	99	10	3.6	695	3,479
	2011	549,318	214	20.4	2,335	24	9	4.2	914	5,463
	2012	563,525	153	36.0	3,367	87	10	6.5	1,673	8,275
	2013	577,892	242	31.2	2,651	36	4	1.7	1,435	10,913
	2014	592,322	264	28.3	2,243	27	14	5.3	760	5,213
	2015	606,961	265	20.5	2,061	35	3	1.1	672	7,864
	2016	623,788	270	20.5	2,104	41	6	2.2	1,206	7,678
**LUNG**										
	2008	491,853	609	1.8	1,867	43	15	2.5	593	–
	2009	511,366	677	16.1	56,215	215	79	11.7	3,787	436
	2010	469,691	667	24.5	30,622	237	89	13.3	4,346	4,221
	2011	549,318	577	22.0	20,868	265	45	7.8	3,473	13,021
	2012	563,525	547	37.8	25,605	1,246	54	9.9	8,152	18,799
	2013	577,892	899	32.0	22,350	949	48	5.3	6,909	8,423
	2014	592,322	984	28.6	19,258	633	71	7.2	6,144	4,868
	2015	606,961	1070	21.9	19,441	695	55	5.1	5,410	37,498
	2016	623,788	923	21.2	19,473	1,152	45	4.9	5,965	36,105
**CERVIX UTERI**^†^										
	2008	245,435	44	16.8	2,727	60	1	2.3	4,810	–
	2009	255,753	67	18.8	15,309	134	13	19.4	3,261	6,322
	2010	266,289	66	23.9	53,763	1,439	16	24.2	691	8,948
	2011	275,492	62	21.4	39,001	3,379	10	16.1	1,338	3,430
	2012	282,763	79	29.3	48,349	4,485	22	27.8	1,506	4,110
	2013	290,167	74	25.3	41,384	1,736	22	29.7	1,767	5,839
	2014	297,616	124	23.6	45,698	2,132	42	33.9	1,730	7,544
	2015	305,206	140	14.8	29,018	1,364	27	19.3	2,591	18,757
	2016	314,008	145	18.2	41,706	1,374	50	34.5	2,909	20,430
**BREAST**^†^										
	2008	245,435	333	16.9	6,407	1,796	24	7.2	2,799	1,862
	2009	255,753	318	18.3	10,258	2,493	21	6.6	5,683	9,196
	2010	266,289	356	25.2	58,237	6,841	47	13.2	3,930	15,329
	2011	275,492	361	21.7	45,393	7,804	62	17.2	1,602	11,839
	2012	282,763	371	28.5	47,580	4,585	58	15.6	2,340	12,531
	2013	290,167	380	23.4	40,154	1,976	41	10.8	3,564	16,601
	2014	297,616	413	21.7	44,402	1,424	50	12.1	979	16,526
	2015	305,206	362	13.5	27,876	1,035	27	7.5	1,006	23,522
	2016	314,008	395	16.9	37,564	1,318	29	7.3	1,501	22,278
Total			22,182				2,948		136,555	

† *Only for women; Data in 2008 were incomplete for cervix uteri cancer*.

A total of 24,278 residents over 40 years old were identified at high risk of colorectal cancer, 13,503 for stomach cancer, 9,988 for liver cancer, 44,779 for lung cancer, 20,603 for cervix uteri cancer, and 23,404 for breast cancer. All these subjects were registered into the high-risk population management database and offered with regular community-based primary care mentioned earlier.

As shown in Table 4, a total of 2,948 cancer cases were detected through the e-HR system, accounting for 13.3% of all 22,182 newly diagnosed cancer cases in Minhang District. The proportions of early-stage cancers through identified e-HR system, both by screening and by subsequent follow-up, were significantly higher than those through regular medical practices (all *p* < 0.0001).

**Table 4:** Comparison of cancer cases detected by e-HR-based programs and by clinical visits in Minhang District of Shanghai, China, 2008–2016.

**Cancer site**	**Identified in cancer screening programs**			**Identified by subsequent follow-up**			**Diagnosed by clinical visits**			***p-*values for *x*^**2**^ tests**
	**No. of cases**	**No. of cases at early stage^†^**	**%**	**No. of cases**	**No. of cases at early stage^†^**	**%**	**No of cases**	**No. of cases at early stage^†^**	**%**	
Colorectum	1,078	311	28.8	1,677	254	15.1	2,401	182	7.6	< 0.0001
Stomach	728	150	20.6	939	135	14.9	2,081	276	13.3	< 0.0001
Liver	79	16	20.3	200	15	7.5	1,902	120	6.3	< 0.0001
Lung	501	79	15.0	1,524	198	13.0	4,928	566	11.5	< 0.0001
Cervix uteri^†^	203	168	82.8	329	264	80.2	269	57	21.2	< 0.0001
Breast^†^	359	182	50.7	871	401	46.0	2,059	681	33.1	< 0.0001
Total	2,948	906	30.7	5,540	1,267	22.9	13,640	1,882	13.8	

† *Cases with TNM staging 0–II for colorectal, gastric, liver, and lung cancers, at stage 0–IIa under FIGO 2,000 classification for cervical cancer, and with TisN0M0/T1N0M0 and primary tumor diameter ≤ 2 cm for breast cancer*.

## Discussion

In Minhang district of Shanghai, China, with over 950,000 residents and over 530,000 residents aged more than 40 years (http://www.shmh.gov.cn/, accessed on Jan 16, 2019), an infrastructure for e-HR-based cancer screening was well-established and a series of screening programs have been implemented effectively to detect major cancers over the past several years, particularly for colorectal cancer, gastric cancer, liver cancer, lung cancer, cervical cancer, and breast cancer. This, to the best of our knowledge, is the first e-HR-based comprehensive health practice in cancer prevention at district/city level, which is regarded as a local level exploration for healthcare reform in China. Currently, the system has achieved a capability of offering relevant services for over 20% of population aged 40 years or above each year.

Comprehensive e-HR systems have been successfully applied in many European countries (16–18) and partly in the United States such as Kaiser Permanente (19) and Veterans Affairs Health Care (20). The successful models of organizing and operating e-HR systems provide platforms for cancer screening in resource-rich settings (21–25). In China, a middle-income country, the e-HR system has been used to identify patterns of non-communicable diseases (26), evaluate effect of an intervention in patients with chronic obstructive pulmonary disease (27), and improve cardiovascular care and outcome (28). The present program, taking advantages of the e-HR system, successfully identified a higher proportion of early-stage cancers than regular medical services, offering an example of applying the e-HR system as a feasible comprehensive cancer care system in resource-limited settings.

Our results also suggest that the e-HR system in Minhang District is not only a surveillance system but also a useful platform for health education and health promotion. Health education as a primary prevention strategy is delivered to all community participants at the very beginning, followed by disease screening as the secondary prevention and where appropriate, post-treatment follow-up and health management as the tertiary service. Furthermore, the e-HR system seems useful for identifying health needs in local settings. For instance, the present program has reached a fairly comparable coverage for colorectal, breast, and cervical cancers with screening programs in the United States (29), but only limited screening services were provided for liver cancer and lung cancer due to the relatively high cost but low sensitivity of the initial screening tests like AFP test, ultrasonic, and chest X-ray examinations.

Evidently, the e-HR system has the potential to extend the accessibility of healthcare services in the general population by coordinating and integrating various healthcare services efficiently. In this case, health services for cancer screening, from risk evaluation, early detection of cancer cases, to post-treatment follow-up and health management, were provided efficiently by multiple institutes based on the e-HR system.

Experiences and lessons also can be learned from this program for sustaining a public health system, which may balance increasing challenges and health needs. The e-HR system requires a team approach to input, analyze, and implement huge data. The doctors in CHSCs and medical centers act as the driving force behind the system, but advanced practice clinicians, nurses, quality coordinators, information technology support, and many others should collaborate to make it successful. In most developing countries, however, limited investment in public health and shortage of medical resources remain a big issue. However, the investment in e-HR system will save public health resources in the long run. As a typical example, Minhang District is a rapidly developing region with limited public health budget. Local annual budget for public health and disease prevention is only 100 Yuan RMB per person (1 US dollar equals about 6.8 Yuan RMB), which cannot cover a universal screening for all kinds of chronic diseases, including cancer. Based on the e-HR system, however, the present early cancer detection and continuous service program costs only about 10% of the annual public health budget for risk assessment, screening, subsidies for clinical check-ups, and health management for high risk people. With possibly increased public health budget in the coming years in Minhang District, it is very likely and foreseeable to extend the program from cancer to other non-communicable diseases. Moreover, the e-HR data can be accessed and used by any researchers once their applications are approved by local Health Commissions, which provide valuable opportunities for further scientific researches.

There are several limitations of this study. First, we did not compare the characteristics of participants and non-participants of the cancer screening program. The potential differences between the two subpopulations may have biased our results. Second, we did not take the sensitivities and specificities of cancer screening methods used in the population into consideration. Several methods with low validity such as CBE, TTM, AFP, and chest X-ray were used in the program, leading to unnecessary costs. Finally, cost-effective analysis of the program was not conducted due to lack of financial data for e-HR system building, limiting our ability to evaluate the system.

## Conclusions

In conclusion, the e-HR system in Minhang District enables local health institutions to provide integrative and comprehensive health care and management for cancers. The successful application of an e-HR system in cancer prevention and control implies that the system may act as an extendable and sustainable infrastructure for comprehensive health care and services for a broad spectrum of diseases and health events.

## Data Availability

The datasets generated for this study are available on request to the corresponding author.

## Ethics Statement

The study was approved by the Institutional Review Board of Minhang District CDC (NO: EC-P-2012-002). Verbal informed consent was obtained from each participant of the cancer screening program.

## Author Contributions

DH and WX drafted the manuscript. DX and NH conceived and designed the study. DH, WX, HS, and WL made substantial contributions to the study design. DH, JZ, and BY are responsible for study coordination. DH and DX are responsible for data quality control. DH and BY are responsible for data wrangling. DH is responsible for data analysis. All authors contributed to the revision of the manuscript and approved the final manuscript.

### Conflict of Interest Statement

The authors declare that the research was conducted in the absence of any commercial or financial relationships that could be construed as a potential conflict of interest.

## Abbreviations

e-HR: electronic health records
CDC: Centers for Disease Control and Prevention
CHSC: Community Healthcare Service Centers
FOBT: fecal occult blood test
RE: rectal exam
TAA: tumor-associated antigens
AFP: alpha-fetoprotein
UT: ultrasonic testing
LDCT: low-dose computerized tomography
CBE: clinical breast examination
TTM: thermal texture maps
MAM: mammography.
